# Association between high-fat diet feeding and male fertility in high reproductive performance mice

**DOI:** 10.1038/s41598-019-54799-3

**Published:** 2019-12-06

**Authors:** M. D. Gómez-Elías, T. S. Rainero Cáceres, M. M. Giaccagli, V. A. Guazzone, G. N. Dalton, A. De Siervi, P. S. Cuasnicú, D. J. Cohen, V. G. Da Ros

**Affiliations:** 1 Laboratorio de Mecanismos Moleculares de la Fertilización, Instituto de Biología y Medicina Experimental (IByME-CONICET), Ciudad Autónoma de Buenos Aires, Buenos Aires, Argentina; 2 Universidad de Buenos Aires. Facultad de Medicina. Departamento de Biología Celular e Histología/Unidad Académica II. Ciudad Autónoma de Buenos Aires, Buenos Aires, Argentina; 3 CONICET-Universidad de Buenos Aires, Instituto de Investigaciones Biomédicas (INBIOMED). Facultad de Medicina. Ciudad Autónoma de Buenos Aires, Buenos Aires, Argentina; 4 Laboratorio de Oncología Molecular y Nuevos Blancos Terapéuticos, Instituto de Biología y Medicina Experimental (IByME-CONICET), Ciudad Autónoma de Buenos Aires, Buenos Aires, Argentina

**Keywords:** Cell biology, Disease model, Metabolic disorders

## Abstract

The increasing worldwide prevalence of metabolic syndrome (MetS), especially in younger populations, is a risk factor for fertility disorders. However, a direct correlation of MetS with male infertility still remains unclear. In this work, we evaluated whether MetS has a negative impact on fertility of hybrid male mice with high reproductive performance. To induce a MetS-like condition, (C57BL/6xBALB/c) F1 male mice were fed a high-fat diet (HFD, 30% fat) for 19 weeks, while controls received a normal-fat diet (NFD, 6% fat). HFD-fed animals exhibited increased body weight, hypercholesterolemia, hyperglycemia and glucose intolerance. *In vivo* fertilisation assays performed along the treatment period showed no differences in fertilisation nor *in vitro* embryo development rates between groups. While testicular weight and morphology were similar in both groups, HFD-fed mice presented lighter epididymides and higher amounts of gonadal fat. Moreover, sperm count was lower in HFD-fed mice, despite normal sperm viability, morphology, motility or acrosome reaction. Finally, no differences were observed in *in vitro* fertilisation rates between groups. In summary, although HFD feeding altered some reproductive parameters, it did not impair male fertility in high performance breeders suggesting the possibility that a fertility impairment could be the result of the cumulative combination of environmental and/or genetic factors.

## Introduction

Infertility is a global health problem affecting 10–15% of couples in reproductive age^1^. While in most of the cases its etiology can be attributed to female or male factors, almost 20% of them correspond to idiopathic infertility. In particular, the evaluation and classification of male infertility relies on a semen analysis including three parameters: concentration, morphology and motility, which have limitations as predictors of sperm fertilising ability^2^. In addition, there is growing evidence supporting that lifestyle factors can affect male fertility through alterations in endocrine profiles, spermatogenesis and sperm function^3,4^. Thus, the identification of the factors contributing to infertility may be critical to offer simpler and/or more effective therapeutic options than the general spectrum of available treatments.

Metabolic syndrome (MetS) has been defined as a set of physiopathological disorders comprising the increase of at least three of the following parameters: abdominal obesity, blood pressure, triglycerides, cholesterol and fasting glucose^5^. In the last 40–50 years, its prevalence has increased in epidemic proportions, affecting 20–25% of the general population^6^. With the increasing number of children, adolescents and young adults dealing with this metabolic disorder, the risk to acquire associated diseases may become a serious health problem in the coming years^7^. In particular, the acceleration in the onset of MetS makes it coincident with the reproductive age, being able to affect any of the multiple processes involved in reproduction (i.e. hormone production, gametogenesis, fertilisation and embryo development), thus compromising the possibility of a couple to achieve pregnancy either by direct effects on reproductive organs or cells, or by indirect effects through other systems.

Given the complex interactions between obesity, dyslipidemia, hyperinsulinemia and the reproductive axis^8^, the effect of MetS as a clinical entity has been less studied than each of its separated components in relation to the fertility of the affected individuals. Despite its relevance, the effect of MetS on male reproductive function has emerged as a critical topic of study only recently. MetS may affect male fertility through several mechanisms including endocrine system regulation, scrotal temperature elevation, oxidative stress and alteration of erectile and ejaculatory functions, which can impair sperm production and/or function^9^. However, the information obtained so far is controversial^10–15^ probably due to the multifactorial nature of this syndrome in addition to the presence of both external (smoking, drugs, stress, etc.) and/or genetic intrinsic susceptibility factors that can affect fertility. In addition, the impact of these factors on fertility is uncertain, as most studies only examine semen parameters rather than sperm function. Therefore, the development of animal models by chronically feeding them high-fat diets to mimic MetS has resulted in a very useful tool for this type of study^7,16^. In this sense, a recently published systematic meta-analysis of the effect of high-fat diets on sperm traits and/or male fertility in different animal models has shown an association between obesity and male fertility without evaluating other MetS features^17^. In the case of the mouse model, most of the studies published so far and included in this meta-analysis have been performed in C57BL/6 mice which are poor reproducers^18^. In this regard, it has been described that multifactorial traits could be attributable to susceptibility genes modulated by the genetic background^19^. Therefore, with the purpose of dissecting the low reproductive ability of that animal model from the actual impact of MetS on fertility, we wondered whether the acquisition of a metabolic disorder could affect the fertility of males from a mouse strain without preexisting reproductive deficiencies. In view of this, in the present study, we evaluated the fertility and sperm functionality of a hybrid mouse strain with high reproductive performance fed a high-fat diet as an experimental MetS model.

## Results

### Generation of a MetS-like condition in B6CF1 male mice

**Figure 1:**
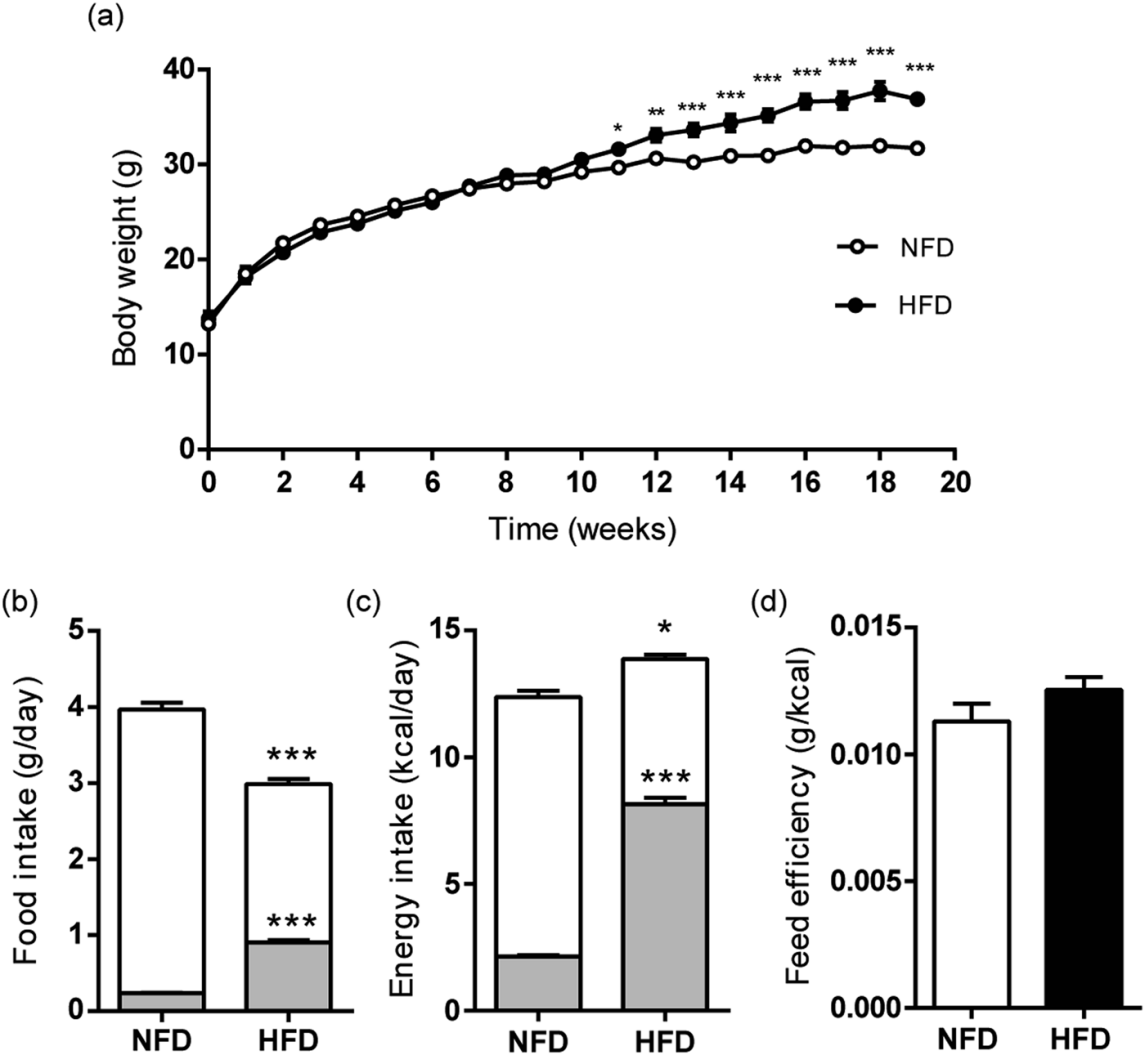
HFD feeding in B6CF1 male mice. (**a**) Weekly average body weight of male mice in NFD and HFD groups; (**b**) daily food intake (white bars) and consumed fat (grey bars); (**c**) daily energy intake (white bars) and energy provided by fat (grey bars); (**d**) feed efficiency (grams of weight gain per energy intake unit). Data are expressed as mean ± SEM. n_NFD_ = 9, n_HFD_ = 9. *p < 0.05, **p < 0.01, ***p < 0.001.

High-fat diet (HFD) fed B6CF1 male mice gained more weight than age-matched normal-fat diet (NFD)-fed controls, resulting in a significantly higher body weight at week 11 that persists up to week 19 (Fig. 1a). Both total food and fat intakes showed significant differences between the control and treated groups: total food intake was higher in the NFD group of animals whereas fat intake was higher in the HFD group (Fig. 1b). As a consequence, energy intake was significantly higher in HFD-fed males (Fig. 1c). Considering that feed efficiency was similar in both cases (Fig. 1d), these results support that body weight gain in HFD-fed animals could be attributed to a higher fat intake.

**Figure 2:**
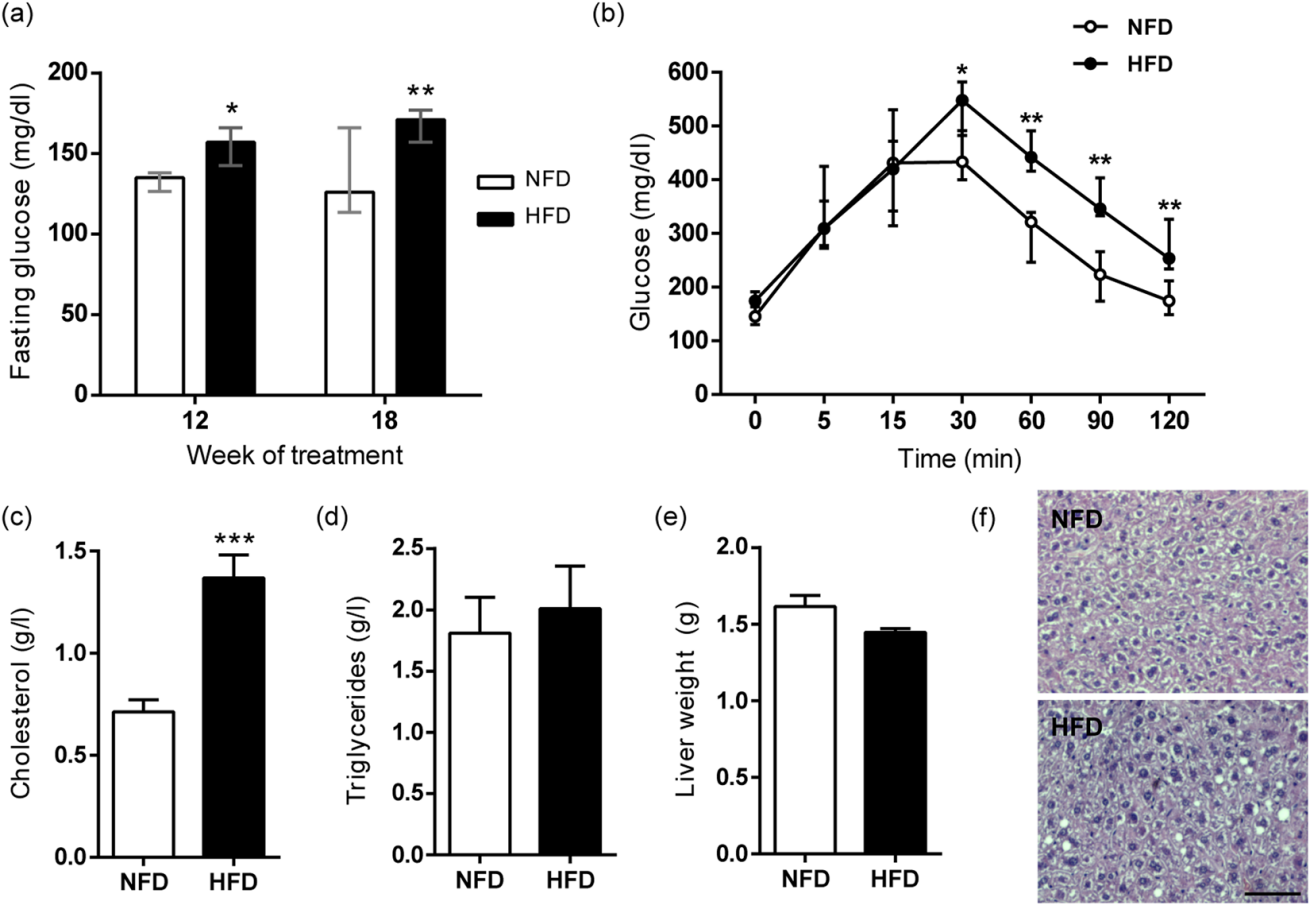
Effect of HFD consumption on metabolic parameters of B6CF1 male mice. (**a**) Fasting glucose levels were determined at week 12 and 18 (n_NFD_ = 9, n_HFD_ = 9). (**b**) Glucose tolerance test (GTT) was performed in week 18 after i.p glucose administration (2 g/kg body weight) after 6 h of fasting (n_NFD_ = 5, n_HFD_ = 6). (**c**) Serum cholesterol and (**d**) triglycerides levels were determined after euthanasia (n_NFD_ = 9, n_HFD_ = 9). (**e**) Mean weight of livers from NFD- and HFD-fed male mice (n_NFD_ = 4, n_HFD_ = 3). (**f**) Representative micrographs of Hematoxylin and Eosin stained liver sections from NFD- and HFD-fed male mice. Bar = 50 µm. Data are expressed as median with interquartile range for (**a,b**), and mean ± SEM for (**c–e**). *p < 0.05, **p < 0.01, ***p < 0.001.

To evaluate the metabolic condition of treated animals, fasting blood glucose was measured at weeks 12 and 18 of treatment. In both cases, significantly higher values were observed for HFD than NFD group (Fig. 2a). In order to obtain additional information regarding the glucose metabolism, glucose tolerance test (GTT) was performed at week 18. Contrary to the males belonging to the NFD group, HFD-fed animals showed higher blood glucose levels 30 min after glucose injection, and a different subsequent response that did not reach the basal levels at the end of the study (Fig. 2b). As a consequence, there was also a significant difference between the area under the curve (AUC) of NFD and HFD groups (NFD: (3.6 ± 0.3) × 10^4^ vs HFD: (4.9 ± 0.3) × 10^4^, p < 0.01).

Different metabolic parameters were also determined at the moment of euthanasia. HFD-fed males presented significantly higher levels of serum cholesterol than those corresponding to the control group (Fig. 2c), and there was no difference between the two groups in either serum triglyceride concentration (Fig. 2d) or liver weight (Fig. 2e). Liver histopathological analysis showed that HFD-fed animals did not develop global steatosis although some cytoplasmic vacuoles were observed (Fig. 2f). Therefore, the increased body weight, hypercholesterolemia and hyperglycemia in HFD-fed animals were consistent with the development of a MetS-like condition.

### Effect of a HFD on *in vivo* fertilisation of B6CF1 male mice

To assess the impact of the HFD on the fertility of B6CF1 male mice, *in vivo* fertilisation studies were carried out at treatment weeks 12, 16 and 18. Pre-pubertal superovulated females were used as a way to challenge sperm to a high number of ovulated eggs allowing the detection of deficiencies in sperm fertilising ability not evident under regular mating conditions^20^. Mating success, measured as the proportion of females presenting copulatory plug after being caged with a male the night of hCG administration, showed no differences between NFD and HFD groups (Table 1). In addition, similar *in vivo* fertilisation rates were observed for both groups at all the evaluated time points (Table 1). *In vitro* development to the blastocyst stage of *in vivo* fertilised eggs was also recorded, without observing significant differences between groups (Table 1).

**Table 1:** Effects of HFD consumption in *in vivo* fertilisation and *in vitro* embryo development.

Group	Mating success*	*In vivo* Fertilisation rate (%)			*In vitro* Embryo development rate (%)		
		week 12	week 16	week 18	week 12	week 16	week 18
NFD	21/23	81.0 ± 6.9 (n = 7)	77.1 ± 7.8 (n = 7)	67.1 ± 9.9 (n = 7)	87.2 ± 4.7 (n = 7)	83.5 ± 3.3 (n = 7)	85.7 ± 6.5 (n = 6)
HFD	21/24	73.6 ± 7.1 (n = 6)	76.9 ± 6.1 (n = 7)	85.1 ± 5.1 (n = 8)	85.0 ± 5.3 (n = 6)	90.1 ± 3.0 (n = 7)	84.0 ± 5.2 (n = 7)

*No. of females with copulatory plug/total number of caged females.

### Effect of a HFD on *in vitro* sperm analysis of B6CF1 male mice

**Figure 3:**
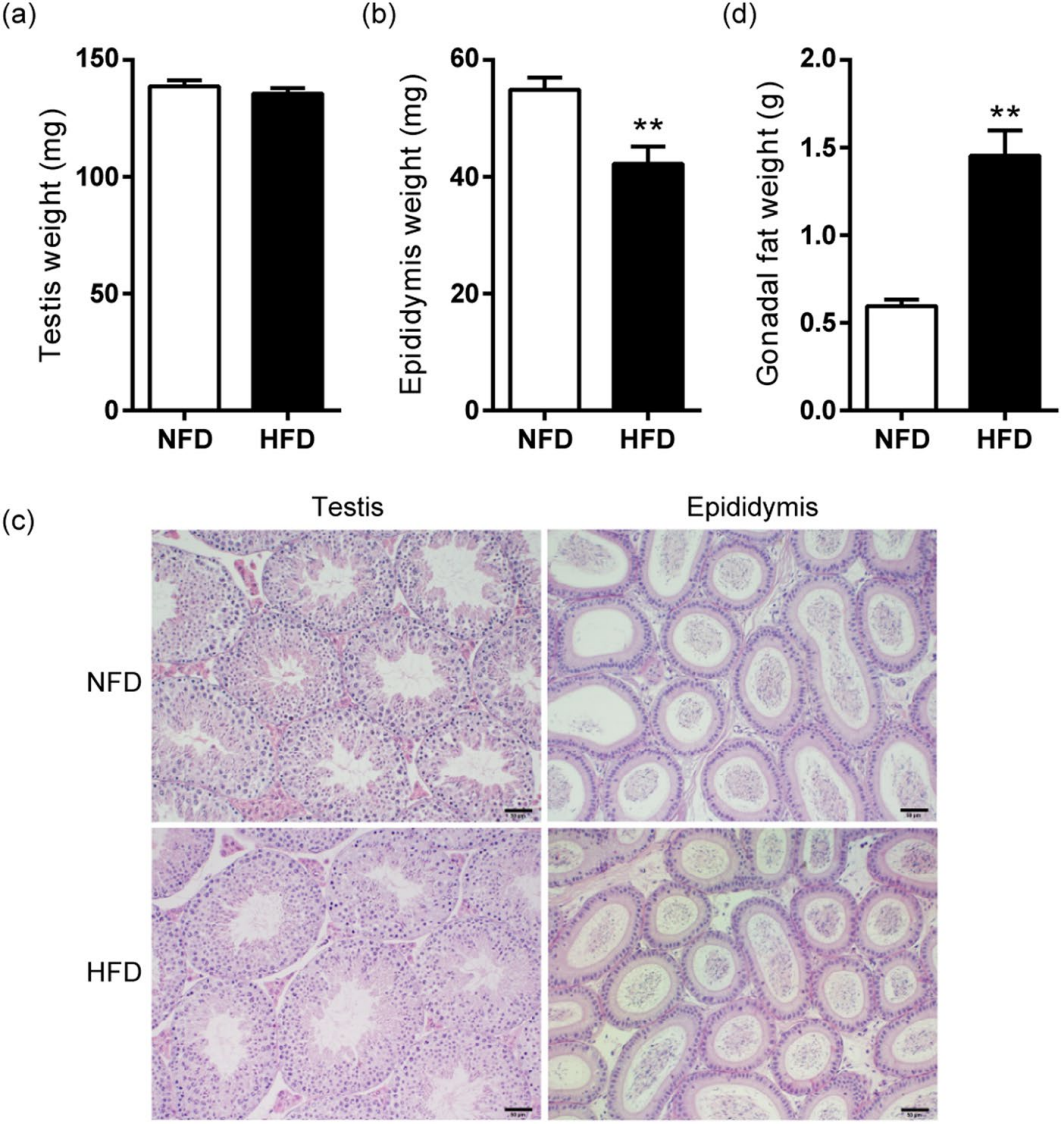
Effects of HFD consumption in reproductive organs of B6CF1 male mice. (**a**) Mean weight of testicles (n = 8) and (**b**) epididymides (n_NFD_ = 7, n_HFD_ = 9). (**c**) Representative micrographs of Hematoxylin and Eosin stained testicular and caput epididymal sections. Bar = 50 µm. (**d**) Mean weight of gonadal fat (n_NFD_ = 4, n_HFD_ = 3). Data are expressed as mean ± SEM. **p < 0.01.

Sperm from HFD-fed mice were subjected to *in vitro* analysis for evaluation of potential differences that could have been overcome under *in vivo* conditions. Testicular weight was similar in HFD- and NFD-fed animals (Fig. 3a). Therefore, HFD-fed mice presented a significant decrease in testicular/body weight ratio compared to controls (NFD: 0.439 ± 0.006% vs HFD: 0.37 ± 0.01%, p < 0.001). By contrast, a decreased epididymal weight was observed in HFD animals (Fig. 3b) with a consequent significant difference in epididymal/body weight ratio (NFD: 0.174 ± 0.006% vs HFD: 0.114 ± 0.008%, p < 0.001). No pathological alterations were found after histological examination of testes and epididymides of HFD- and NFD-fed animals (Fig. 3c). In addition, gonadal fat weight was significantly higher in HFD-fed mice compared to the control group (Fig. 3d).

**Figure 4:**
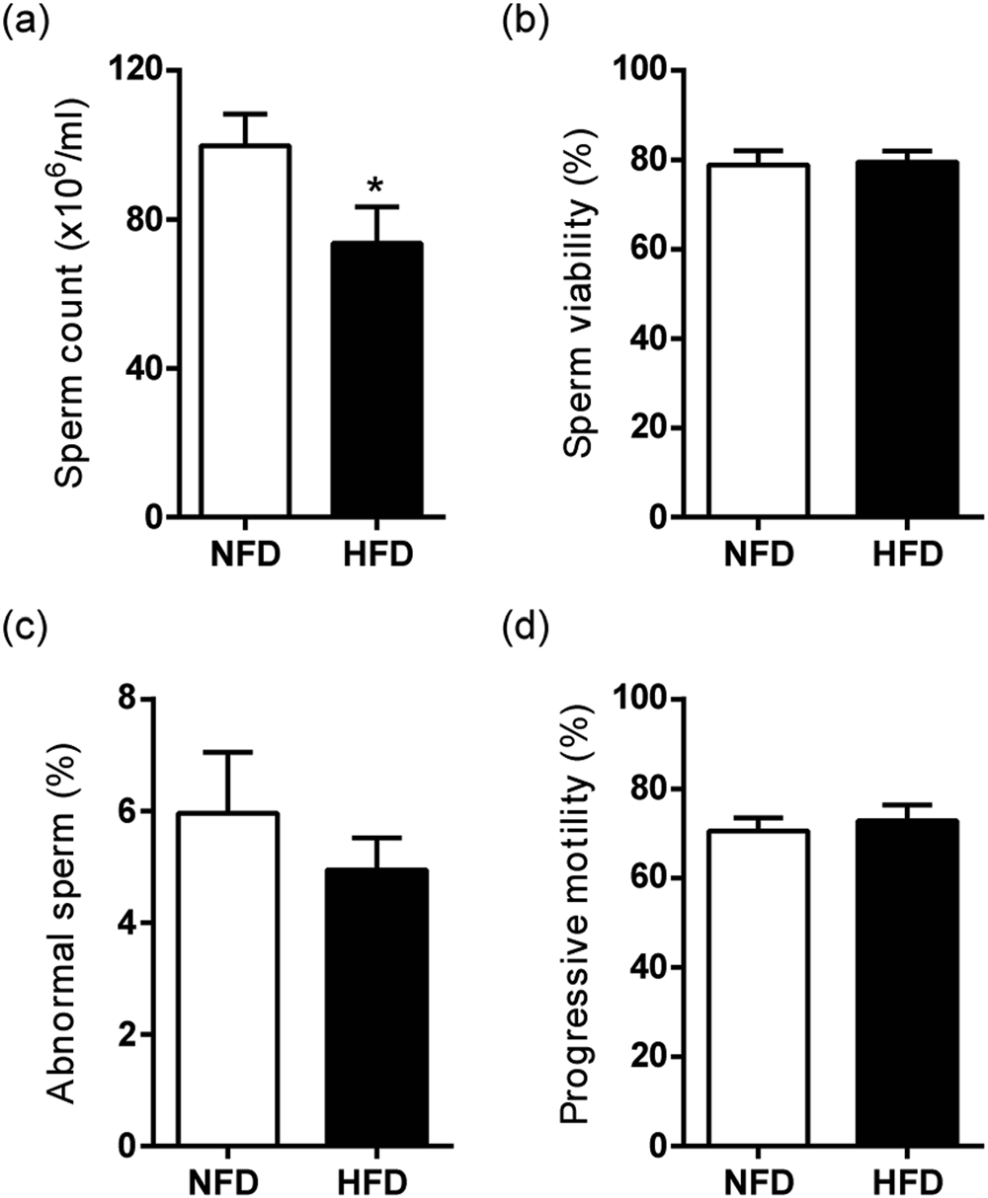
Effects of HFD consumption in sperm parameters of B6CF1 male mice. (**a**) Sperm count, (**b**) sperm viability, (**c**) percentage of abnormal sperm and (**d**) progressive motility in NFD- and HFD-fed mice (n_NFD_ = 9, n_HFD_ = 9). Data are expressed as mean ± SEM. *p < 0.05.

**Figure 5:**
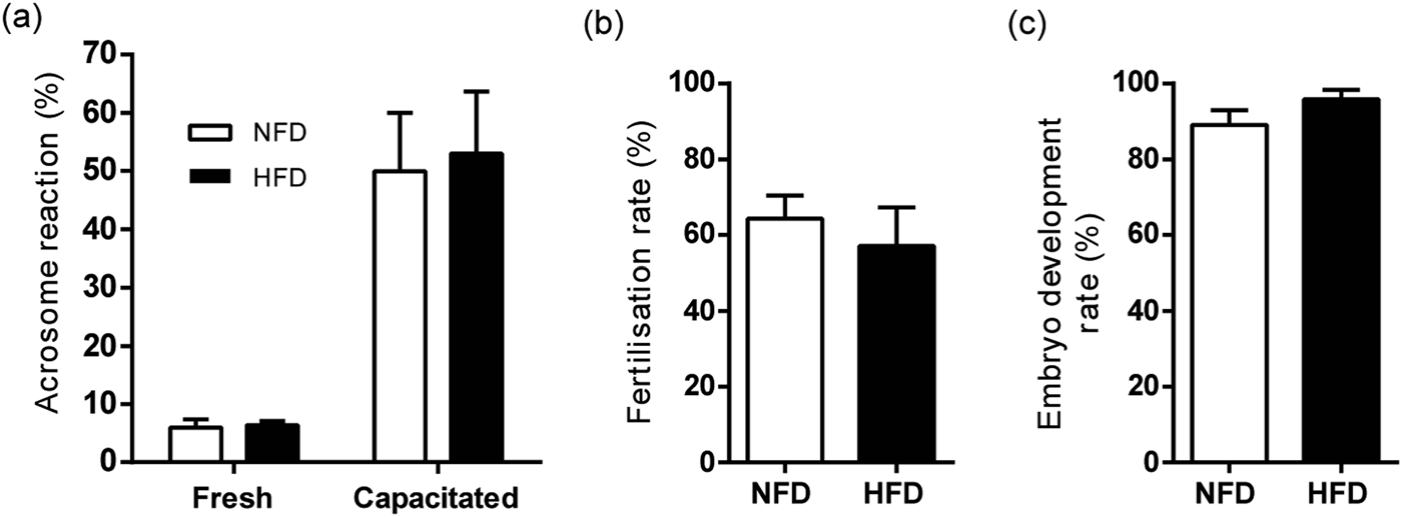
Effects of HFD consumption in sperm fertilising ability of B6CF1 male mice. (**a**) Percentage of acrosome reaction before (fresh) and after capacitation (capacitated) (n_NFD_ = 7, n_HFD_ = 7), (**b**) *in vitro* fertilisation rate (n_NFD_ = 8, n_HFD_ = 8) and (**c**) *in vitro* embryo development rate (n_NFD_ = 6, n_HFD_ = 6) from NFD- and HFD-fed mice. Data are expressed as mean ± SEM.

Sperm analysis showed significant differences between HFD- and NFD-fed mice in cauda epididymal sperm count (Fig. 4a), whereas similar values were observed in both groups for sperm viability (Fig. 4b), morphology (Fig. 4c) and progressive motility (Fig. 4d). After *in vitro* capacitation, sperm from both groups exhibited a comparable ability to undergo spontaneous acrosome reaction (Fig. 5a). Finally, *in vitro* fertilisation (Fig. 5b) and early embryo development rates (Fig. 5c) showed no difference between groups. In summary, the functional experiments showed that, although presenting lower sperm count, HFD-fed B6CF1 male mice did not exhibit alterations in sperm fertilising ability either *in vivo* or *in vitro*.

## Discussion

In the current study, we explored the impact of HFD-induced MetS-like condition on male fertility and sperm functionality of mice with high reproductive performance. HFD-fed B6CF1 animals developed a physiological condition compatible with MetS that included significant rises in body weight, fasting glucose and cholesterol levels, as well as glucose intolerance. Our primary finding is that this metabolic condition did not impact on the reproductive performance of hybrid B6CF1 animals, albeit a significant decrease in sperm count.

The fact that the HFD-challenged B6CF1 males were fertile is in accordance with the growing list of conflicting data around the effects of MetS on male reproduction in rodents and humans^10–15,17^. This controversy is not surprising given that metabolic diseases are a combination of genetic and environmental factors, and its impact on fertility may be different between individuals. Among the factors that might confound the results are the composition and/or characteristics of the HFD used in each study with a direct consequence in the development of MetS. Moreover, it has been suggested that the effects of dietary fat on health and disease are likely to depend on the balance of other macro and micronutrients^21,22^. In this sense, we found some differences in body weight gain and food consumption between our study, using a prepared diet, and others using commercial ones. Interestingly, whereas in our experimental conditions the significant difference in body weight between the two groups was reached after 11 weeks of treatment, other studies achieved weight differences before 6 weeks of high-fat feeding^23–28^. This delay in reaching differences in body weight might be due to the reduced food intake of HFD-fed mice in our study with a consequent energy intake only 12% higher than NFD controls, in contrast to the 55% and 20% increases reported by others^26,27^. In addition, examination of the livers showed that the HFD treatment did not produce steatosis or increases in the organ size in B6CF1 males as observed in other mouse models^23,25,29,30^, that could be a consequence of the aforementioned gradual intake of excessive fat. In this regard, considering that gut microbiota has emerged recently as being crucial in the etiology of metabolic diseases (reviewed in^31^), the possibility that in our study the MetS-like condition was attenuated by the local microbiota cannot be ruled out.

Considering the multifactorial nature of MetS, the use of different mouse strains could contribute to a better understanding of the association between the metabolic disorder and fertility. Differently to the well-established C57BL/6 mouse model of HDF-induced MetS^17,27,32^, the B6CF1 strain has not been studied before and may exhibit a different susceptibility to HFD with different consequences on the reproductive phenotype. The fact that nowadays metabolic diseases are observed from an early age and could result in possible reproductive deficiencies in the future drives the development of new experimental models that better reflect the human situation. In this sense, the power of using the B6CF1 model relies on the hybrid vigor of the animals, with high number of pups per litter and good sperm quality, making this model more suitable than C57BL/6 to mimic diet-induced MetS in young men. In addition, the lack of preexisting fertility deficiencies in these B6CF1 animals allowed us to study the actual impact of MetS on fertility. Our results showed that the high reproductive performance of B6CF1 males was not affected by HDF-induced MetS. Neither mating success, an indirect indicator of testosterone levels, nor *in vivo* sperm fertilising ability was changed after the HFD challenge, arguing against a robust causal effect of high-fat feeding on male infertility. In addition, *in vitro* early embryo development was not impaired in our model, although the possibility of paternal epigenetic inheritance of metabolic disorders due to HFD-induced paternal obesity (reviewed in^33^) requires further investigation. Altogether, the observed lack of fertility phenotype could be a consequence of the intrinsic reproductive potential of the B6CF1 animals and/or the gradual acquisition of MetS in our model. Therefore, the way in which the metabolic disorder develops in each individual could be one of the causes of the variability reported for the association between metabolic and fertility disorders in men.

HFD-fed males exhibited a higher amount of gonadal fat, proposed to increase testicular and epididymal temperature, thus affecting sperm production, maturation and storage^34^. In this sense, we observed a reduction in sperm count, similar to previous reports^28,35,36^ and opposed to others^23,26,37,38^. In addition, differently from other studies reporting deleterious effects of HFD on testicular morphological structure^25,28–30,35,36^, our histological analysis showed no major differences in this feature between groups, resulting in normal spermatogenesis and sperm morphology in the HFD group. However, HFD-fed mice exhibited a decrease in epididymal weight, consistent with the lower epididymal sperm count. Although these reductions could be due to a decrease in testosterone levels, normal testis histology, copulatory behaviour and fertility in HFD-fed mice set aside a negative effect of this hormone on our model. Despite the fact that the most reported alteration in sperm from HFD-fed males turns out to be the decrease in motility^23–25,37–40^, we did not observe this deficiency in our animals. In the case of the mouse, as mentioned above, reports in the literature were principally performed in C57BL/6 background, different to the hybrid B6CF1 used here. In the overall, among all the analysed sperm parameters, only epididymal sperm concentration was reduced in B6CF1 animals. However, it did not produce any impact on fertility, in accordance with previous results in another mouse model of sperm count reduction by unilateral vasectomy^20^. If this data could be extrapolated to humans in which sperm concentration remains a cornerstone of male fertility evaluation^2^, additional sperm functional tests should be considered as better prognostic indicators.

In summary, although HFD feeding altered some reproductive parameters, it did not compromise male fertility in mice without preexisting reproductive deficiencies. Our findings suggest the possibility that fertility impairment in humans could be the result of a combination of different environmental and genetic factors that may act in a cumulative manner with other predisposing factors from any of the two members of the couple. It is important to mention that patients who consult for infertility, in general, are not evaluated in terms of their metabolic status. Therefore, the study of the metabolic status of each individual could offer an alternative treatment to assisted reproductive techniques (ART), for example through personalised nutrition, rendering reproductive benefits.

## Methods

### Ethical approval

Approval for the study protocol was obtained from the Institutional Animal Care and Use Committee of Instituto de Biología y Medicina Experimental (No. 22/2017). Experiments involving animals were performed in accordance with the Guide for Care and Use of Laboratory Animals published by the National Institutes of Health.

### Animals and reagents

Hybrid (C57BL/6xBALB/c)F1 (B6CF1) mice were housed in the animal facility at IBYME-CONICET (Buenos Aires, Argentina) and maintained with food and water *ad libitum* in a temperature-controlled room (23 °C) with light:dark (12:12 h, lights on: 7:00 AM) cycle. All reagents and chemicals were of molecular biology grade and were purchased from Sigma-Aldrich Chemicals (St Louis, MO, USA), unless otherwise specified.

### Generation of a murine model of MetS-like condition

Three-week-old male mice (body weight: 13 ± 2 g) were individually caged and randomly allocated to receive a control normal-fat diet (NFD, n = 9) or a high-fat diet (HFD, n = 9) for 19 weeks. NFD consisted of regular chow food containing (in weight) 6.0% fat, 24.0% protein and 40.5% carbohydrate (3210 kcal/kg) (GEPSA, Buenos Aires, Argentina). HFD was prepared by supplementation of NFD with lard (Hebos SA, Buenos Aires, Argentina) as previously described^41^, resulting in 30.3% fat, 17.8% protein and 30.0% carbohydrate (4640 kcal/kg). The composition of these macronutrients in the prepared HFD is comparable to that reported by others in fertility studies^23,25,42^. Body and food weights were recorded weekly.

### Measurement of metabolic parameters

Fasting blood glucose levels were determined in a sample collected from the tail vein after 6 h of fasting using a glucometer (Accu-chek active, Roche, Argentina). At the moment of euthanasia, blood was drawn by direct heart puncture, and serum was separated to measure cholesterol and triglycerides levels with specific kits (Colestat enzimático and TG-Color, respectively; Wiener lab, Rosario, Argentina).

### Glucose Tolerance Test (GTT)

GTT was performed at week 18 after 6 h of fasting by intraperitoneal (i.p.) administration of 2 g/kg body weight glucose (40% w/v in saline solution). Glucose concentration was measured using a glucometer as described before at time points 0 (prior to glucose administration), 15, 30, 60, and 120 min.

### *In vivo* fertilisation assays

*In vivo* fertilisation assays were performed as previously described^43^. Briefly, immature females were superovulated by an injection (i.p.) of equine chorionic gonadotropin (eCG; 5UI; 4 h before lights turn out; Syntex, Buenos Aires, Argentina), followed by the administration (i.p.) of human chorionic gonadotropin (hCG; 5UI; 6 h before lights turn out; Syntex) 46 h later. After hCG administration each female was caged with a NFD- or HFD-fed male for one night, and successful mating was confirmed by the presence of a copulatory plug the following morning (day 0). Eggs were then recovered from the oviducts and transferred to 50 µl drops of fresh KSOM medium at 37 °C in a 5% (v/v) CO_2_ atmosphere in air. The percentage of 2-cell embryos on day 1, as an indicator of fertilisation, and the percentage of embryos in blastocyst stage on day 4 were determined.

### Sperm capacitation

Mouse spermatozoa were recovered by incising the cauda epididymides in 300 µl capacitation medium^44^ supplemented with 0.3% (w/v) bovine serum albumin (BSA) under paraffin oil (Ewe, Sanitas SA, Buenos Aires, Argentina) (“swim-out”). Aliquots of the suspension were added to 300 μl fresh medium previously placed in tissue culture dishes to give a final concentration of 5–10 × 10^6^ cells/ml. Sperm suspensions were then incubated for 90 min under paraffin oil at 37 °C in an atmosphere of 5% (v/v) CO_2_ in air.

### Sperm parameters determination

To evaluate morphology and motility, sperm suspensions were placed on pre-warmed slides and analyzed subjectively under a light microscope (400×). We considered progressive motile sperm those cells that moved in a forward direction. We did not include in this category those sperm that vibrated or rotated in the same place. For sperm count, after swim-out, sperm suspensions were diluted in water to prevent sperm movement, and the number of sperm heads was recorded by a standard method using a Neubauer Chamber under a light microscope (400×). Sperm viability was assessed by dye exclusion using 0.5% w/v Eosin Y (Sigma), and the percentage of spermatozoa that did not incorporate the dye (i.e. viable spermatozoa) was determined employing a light microscope (400×).

### Histological analysis

Liver, testes and epididymides were dissected, weighed and fixed for at least 24 h by immersion in 4% formaldehyde in phosphate buffered saline (livers) or Bouin solution (testes and epididymides). Tissues were then processed for paraffin embedding and sectioning by routine methods. Samples were stained with Hematoxylin and Eosin using standardised protocols and examined by light microscopy (100× or 400×).

### Acrosome reaction assays

The acrosomal status of capacitated sperm was evaluated by Coomassie Brilliant Blue staining as previously described^45^. At least 400 spermatozoa were evaluated in each treatment slide in a light microscope (400×) and the percentage of spermatozoa with a reacted acrosome was calculated.

### Egg collection and *in vitro* fertilisation (IVF) assays

*In vitro* fertilisation was performed as previously described^46^. Briefly, female mice were superovulated by an injection of eCG, followed by hCG 48 h later. Egg-cumulus complexes were collected from the oviducts 13–14 h after hCG administration and pooled. Cumulus-intact eggs were inseminated with capacitated spermatozoa (final concentration 1–5 × 10^5^ spermatozoa/ml) and gametes were co-incubated for 5 h at 37 °C in an atmosphere of 5% (v/v) CO_2_ in air. Eggs were then transferred to fresh capacitation medium and 15 h later the percentage of two-cell embryos was recorded. Embryos were then transferred to fresh KSOM medium to evaluate the development to blastocyst stage as described before.

### Statistical analysis

Statistical analyses were performed with GraphPad Prism Software (San Diego, CA, USA). For percentage data, arcsine transformation was performed before submitting them to statistical analysis. Shapiro-Wilk normality test was conducted for each set of values and data was considered not normal at p < 0.10. Homoscedasticity was evaluated by F test and in all cases variances were not significantly different between groups (p > 0.05). Statistical significance of the data that followed a normal distribution was analyzed with Student´s *t*-test (for food and energy intake, feed efficiency, cholesterol and triglycerides levels, organs’ weights, sperm count, viability, morphology, motility, acrosome reaction, and fertilisation and embryo development rates) or two-way analysis of variance (ANOVA) (for body weight over time). In these cases, values were presented as mean ± standard error of the mean (SEM). In the case of not normal distributions, statistical significance was analyzed with Mann-Whitney U test (for glucose determinations), and values were presented as median with interquartile range. In all cases, significance was established at p < 0.05.

## Data Availability

All data generated or analysed during this study are included in this published article.
